# Comparative transcriptome analysis of basal and zygote-located tip regions of peanut ovaries provides insight into the mechanism of light regulation in peanut embryo and pod development

**DOI:** 10.1186/s12864-016-2857-1

**Published:** 2016-08-11

**Authors:** Ye Zhang, Pengfei Wang, Han Xia, Chuanzhi Zhao, Lei Hou, Changsheng Li, Chao Gao, Shuzhen Zhao, Xingjun Wang

**Affiliations:** 1 Life Science College of Shandong University, Jinan, 250100 People’s Republic of China; 2 Biotechnology Research Center, Shandong Academy of Agricultural Sciences, Shandong Provincial Key Laboratory of Crop Genetic Improvement, Ecology and Physiology, Jinan, 250100 People’s Republic of China

**Keywords:** *Arachis hypogaea*, Ovary, Light signaling, Embryo development, Gene expression profiling, Peanut peg

## Abstract

**Background:**

Peanut zygotes typically divide a few times to form a pre-embryo before further embryonic development halts under normal day/night photoperiods. Ovary elongation, however, continuesforming a downward growing peg-like structure. When the peg is buried in the soil, embryo development resumes in the darkness. The embryo-located region (ER) of the peg begins to enlarge and form a pod, while the basal region (BR) of the peg has a distinct fate. The molecular mechanisms governing these unique embryo development processes are unknown.

**Results:**

In this study, histological analysis demonstrated that from 4 days after pollination to 3 days after soil penetration, the peanut pre-embryo remained morphologically similar. By 9 days after soil penetration, the embryo had changed to a globular embryo. Transcriptome analysis revealed differentially expressed genes in the ER and BR before and after peg soil penetration. In addition to light signaling and plant hormone metabolism genes, we identified differentially expressed genes in the ER that contribute to embryo development and pod formation processes, including MADS-box transcription factors, xyloglucan endotransglucosylase/hydrolase protein, cellulose synthase, homeobox-leucine zipper (HD-Zip) protein family genes, amino acid permease, and seed growth and embryo morphogenesis regulators (DA1, TCP3, and YABBY).

**Conclusions:**

A large number of genes were found to be differentially expressed in the ER and BR across three developmental peg stages. Exact changes in gene expression were also identified in the ER during early embryo and pod development. This information provides an expanded knowledgebase for understanding the mechanisms of early peanut pod formation.

**Electronic supplementary material:**

The online version of this article (doi:10.1186/s12864-016-2857-1) contains supplementary material, which is available to authorized users.

## Background

Peanut (*Arachis hypogaea* L.) is one of the most important oil crops worldwide. Their unique geocarpic pod development makes peanuts different from most other legumes. Previous studies have demonstrated that after fertilization, peanut zygotes divide several times, forming a pre-embryo. Further embryonic development is inhibited under light or normal day/night conditions. However, ovary elongation continues due to the activity of an intercalary meristem just behind the pre-embryo [1]. This elongated ovary containing the embryo is usually referred to as the “peg” [2, 3] by peanut growers and researchers, while the term gynophore is primarily used in the literature. A peg cross-section exhibits the typical anatomical characteristics of a dicot stem, while the peg responds positively to gravity and grows like a root. The intercalary meristem of the peg is the site of cell division and is responsible for the elongation of the peg. It is also responsible for sensing and responding to gravity, light, and mechanical stimuli [4–6]. With geotropic growth, the tip region of the peg where the embryo is located is buried beneath the soil. Cell division of the pre-embryo resumes underground in the darkness. After about 9 days, the pre-embryo develops into a globular-stage embryo, and the pod becomes enlarged. Meanwhile, elongation and downward growth of the peg ceases. During this process, the ovary experiences a significant change in environmental conditions, including light signals, mechanical stimuli, moisture, and nutrition [7]. Should the peg fail to penetrate the soil, the normal day/night conditions aboveground inhibit completion of embryo and pod development. Phytochromes (red/far-red light receptors) change significantly before and after peg soil penetration [8, 9], confirming the critical role of light during the transition from peg elongation to embryo and pod development. However, the downstream phytochrome signaling pathway involved in this biological process remains unclear.

Peanut embryo and pod development are a complex process that is regulated by both endogenous and environmental signals. Previous studies have shown that plant hormones, such as auxin, abscisic acid (ABA), kinetin, gibberellins (GA), and ethylene, may play important roles in peanut embryo development and peg elongation [6, 10–13]. For example, our previous research indicated that auxin content decreases significantly after peg soil penetration (unpublished data). Indole-3-acetic acid (IAA) immunolocalization experiments demonstrated that no IAA was present in the unfertilized ovary. However, IAA could be detected in the ovary wall, the epidermis of the elongation zone, the cortex, and the intercalary meristem with peg aerial growth [14]. After soil penetration, IAA was no longer detected at the intercalary meristem region, in accordance with reduced peg elongation. The ABA and gibberellins contents of dark-grown pegs were significantly lower than those of aerial-grown pegs [6]. Dark-grown pegs release twice as much ethylene as aerial-grown pegs [13].

Light signals play a critical role in peg elongation, embryo development, and pod growth. Several studies have shown that light signals control the cessation and reactivation of peanut embryo and pod development in vitro [13, 15–17]. Peanut peg elongation is promoted by light, while pod enlargement is simultaneously inhibited by light [13]. Peg meristems remain active and cause peg elongation as long as they are exposed to light. The ovary does not start to swell until the peg is buried into the soil [15, 18]. In addition, when the peg tip region penetrates the soil, a mechanical stimulation occurs. Earlier studies indicated that this mechanical stimulus was required for normal pod growth and development [15, 19]. However, without the stimulus, the resumption of embryo development could indeed occur. For example, pegs in a solution containing the appropriate nutrients can grow normally and form pods in the dark [7]. Under field-grown conditions, peg soil penetration is indispensable for peanut pod enlargement. However, how environmental signals regulate endogenous biosynthesis and signal transduction pathways and eventually lead to successful embryo and pod development is largely unknown.

Over the last century, a number of physiological and anatomical studies have attempted to address the effects of environmental factors on peanut embryo development and pod swelling [20]. In recent years, with the application of high-throughput sequencing technology, several researchers have used RNA-seq to analyze changes in gene expression that occur during peanut embryo and pod development [21, 22]. DNA microarrays and gene chips have also been used to study gene expression in an attempt to understand the molecular mechanisms that govern peanut embryo and pod development [23, 24]. Proteomic analysis has been used to identify candidate proteins that may play key roles in peg and pod development [25, 26]. These studies identified a range of candidate genes and proteins that may be critical in regulating pod development. In our earlier transcriptome profiling study, we identified a number of genes that were differentially expressed in aerial-grown green pegs, dark-grown white pegs without pod enlargement, and dark-grown pegs carrying very small pods. However, in that study, we used pegs 1–2 cm in length, which contain both the embryo-located tip region and the basal region. We were therefore unable to assess differences in gene expression between these two regions, whose developmental fates are completely different; the tip region develops into a mature pod, while the basal region maintains the peg structure. Separate analyses of gene expression in the tip and basal regions may provide additional understanding of light regulation in peanut pod and embryo development.

In this study, we investigated gene expression in pegs of three developmental stages: (1) green or purple aerial-grown pegs (Stage 1, S1); (2) white pegs that had been buried in the soil for approximately 3 days and in which pod enlargement was not detected (Stage 2, S2); (3) pegs that had been buried in the soil for approximately 9 days and in which pod enlargement had been initiated (Stage 3, S3) [21]. Each peg was divided into two parts, the embryo-located tip region (ER) and the basal region not containing the embryo (BR). We compared the gene expression patterns of these two regions across the three developmental stages of pegs.

## Methods

### Plant materials

Plants derived from Luhua14, a cultivated peanut strain, were grown at the Shandong Academy of Agricultural Sciences experimental farm. Three developmental stages of pegs were used in this study. S1 and S2 pegs were divided into two parts: the 3-mm ER and 10-mm BR. S3 pods were also divided into two part: the enlarged ovary and the remaining 7-mm section next to the enlarged area. Tissues were immediately frozen in liquid nitrogen for RNA extraction. In total, 12 samples were used to detect global changes in gene expression. Two biological replicates were used in this study.

### Paraffin sectioning

S1, S2, and S3 pegs with a length of 8–10 mm were excised from the plants. Younger pegs, 2–3 mm in length, were assigned to Stage 0 (S0) and were harvested 4 days after fertilization while growing upward. Approximately 20 pegs from each stage were used for paraffin sectioning. Samples were fixed immediately in formalin-acetic acid-alcohol (FAA) for 24 h at 4 °C, then washed and dehydrated with a gradient ethanol series (70, 85, 95, and 100 %). After dehydration, the tissues were cleared with xylol, embedded in paraffin, and sectioned into 8–10 μm sections. After drying at 37 °C, sections were de-paraffinized and hydrated in an ethanol gradient series (100, 95, 85, 70, 50, 30 %, and distilled water) before being stained with Toluidine Blue O reagent. After clearing and mounting, sections were observed under a microscope.

### RNA isolation and high-throughput sequencing

Total RNA was extracted from the frozen samples using Trizol Reagent (TaKaRa, Inc., Dalian, China) according to the manufacturer’s instructions. RNA samples were first treated with DNase I to degrade any DNA contamination. RNA quality and purity were detected by Agilent 2100 and NanoDrop. We enriched for mRNA using oligo (dT) magnetic beads, and fragmented the RNA into short (~200 nt) fragments. First-strand cDNA was synthesized using random hexamer primers. Buffer, dNTPs, RNase H, and DNA polymerase I were added to synthesize the second strand. Double-stranded cDNA was purified with magnetic beads. Ends were then repaired, and 3ʹ adenines added. Finally, sequencing adaptors were ligated to the fragments, and fragments were enriched by PCR amplification. An Agilent 2100 Bioanalyzer and ABI StepOnePlus Real-Time PCR system were used to qualify and quantify the sample library. Library products were sequenced (50 bp single reads) using the Illumina HiSeq™ 2000 system. Sequencing data are available at NCBI’s Short Read Archive under accession number SRP064700.

### Digital gene expression profile analysis

Raw reads were generated from each cDNA library. Low-quality reads and adaptor sequences were removed, and clean reads were mapped to the reference transcripts of *Arachis ipaensis* (a progenitor of cultivated peanut, http://peanutbase.org/files/genomes/Arachis_ipaensis/annotation) using SOAP2 and SOAPaligner [27]. Clean reads that uniquely mapped to the reference sequences were included in the final analysis. Statistics and bioinformatics analyses included analysis of the number of clean reads mapped to reference genes and genome, sequencing saturation, and random sequencing distribution. Gene expression levels were calculated based on the number of reads mapped to the reference sequences and then normalized to RPKM (reads per kb per million reads), which is a standard method in gene expression analysis [28]. Pearson’s correlation coefficient for each gene was calculated across two biological replicates. The NOISeq method was used for identifying differentially expressed genes and building a noise distribution model [29]. NOISeq uses a sample’s gene expression in each group to calculate M (the log2 ratio) and D (the value of the absolute difference) of all paired conditions and to build a noise distribution model. For each gene, A, NOISeq computes the average expression in the control group (Control-avg) and the average expression in the treatment group (Treat-avg). Then, the fold-change (M_A_ = log2(Treatment-avg/Control-avg)) and absolute difference value (D_A_ = |Control-avg – Treat-avg|) are determined. If M_A_ and D_A_ diverge markedly from the noise distribution model, gene A is defined as a differentially expressed gene (DEG). If gene A differentially expresses between control group and treatment group, we set G_A_ = 1, otherwise set G_A_ = 0, and give a definition for probability of gene A differentially expressing as following formula:

$$ P\left({G}^A = 1\left|{{\mathrm{x}}^{\mathrm{A}}}_1,\ {{\mathrm{X}}^{\mathrm{A}}}_2\right.\right) = P\left({G}^A = 1\left|{M}^A={m}^a,{D}^A={d}^a\right.\right) = \mathrm{P}\ \left(\left|M*\right|<\left|{m}^a\right|,D*<{d}^a\right) $$

When P is greater than threshold value, its corresponding gene is thought to differentially express between groups. Finally, DEGs were screened according to the following criteria: fold-change ≥ 2 and divergence probability ≥ 0.8. Gene ontology (GO) functional classification of DEGs was performed by WEGO software. Pathway analyses were carried out using KEGG and BLASTX (E value < 0.00001) against the NCBI Nr database.

### Quantitative RT-PCR validation of DEG results

Quantitative RT-PCR (qRT-PCR) was used to verify the transcription levels of 14 randomly selected genes. RNA samples used for qRT-PCR were the same as those used in high-throughput sequencing experiments. Gene-specific primers were designed using Primer Premier 5.0 software and are listed in Additional file 1: Table S1. Each 20 μL qRT-PCR reaction mixture contained 2 μL of 50-fold diluted first-strand cDNA, 0.5 μL of each primer (10 μM), and 10 μL 2X FastStart Universal SYBR Green Master Mix (Roche, USA). An ABI 7500 real-time PCR system was used under the following conditions: 95 °C for 10 min, followed by 40 cycles of 95 °C for 15 s and 60 °C for 1 min. Peanut actin was used as a reference gene for normalizing expression levels. Non-specific products were identified by melting-curve analysis. The Ct value of each gene and RNA-seq results are listed in Additional file 2: Table S2. Relative gene expression levels were determined using the 2^-△△CT^ method, as described [30].

The R package was used for analyzing Pearson’s correlation coefficient (PCC) of quantitative RT-PCR, log2-transformed RPKM values, and RNA-seq results. The maximum expression level of each selected gene was considered to be 100, and the expression levels of the other genes were transformed accordingly. For PCC analysis, the log2-transformed RPKM of gene expression was the average of three technical replicates (with two biological replicates). A heat-map of S3-ER and S3-BR gene expression was generated by Scalable Vector Graphics.

## Results

### Observation of early peanut embryo development

**Fig. 1:**
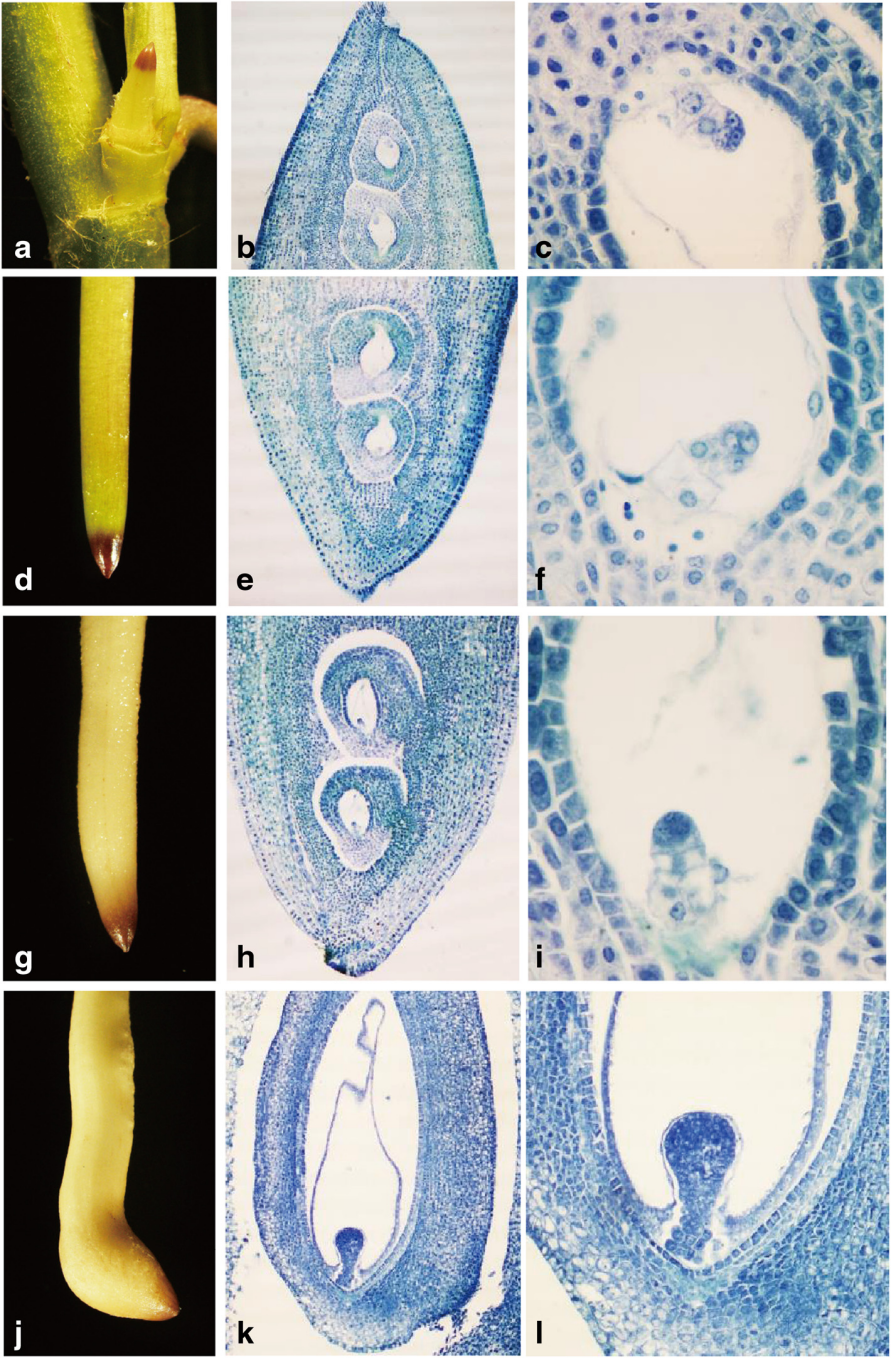
Anatomical analysis of peanut pegs from four developmental stages. **a** Younger pegs with 2–3 mm in length (S0 pegs); **b** and **c** longitudinal section of S0 pegs (amplification: 40× and 400×, respectively); **d** *green* or *purple* aerial-grown pegs (S1 peg); **e** and **f** longitudinal section of S1 peg (amplification: 40× and 400×, respectively); **g** *white* pegs after soil penetration without pod enlargement (S2 peg); **h** and **i** longitudinal section of S2 peg (amplification: 40× and 400×, respectively); **j** pegs after soil penetration and pod enlargement (S3 peg); **k** and **l** longitudinal section of the swelling pod (amplification: 40× and 400×, respectively)

Previous studies indicated that peanut zygotes divide only a few times after fertilization before the developmental process of the embryo ceases. Only when the pegs penetrate into the soil does embryonic development resume. In this study, four developmental stages of pegs were used to study early embryo development (Fig. 1). Histological sections showed two anatropous ovules located to the tip region of the peg. Four days after fertilization, pegs were approximately 2–3 mm in length and growing upward (S0). A rod-like pre-embryo was clearly present in the ovule (Fig. 1b and c). The lengths of aerial-grown (downward) pegs varied significantly, from 5 mm to over 100 mm. The growth periods of these pegs may last anywhere from a few days to more than 2 weeks, depending on their position on the plant. However, the morphology of the pre-embryo at this stage (S1) is similar to that in S0 pegs (Fig. 1e and f). The peg tip grew white in color after being buried into the soil for approximately 3 days (S2). From the sections of S2 pegs, we observed that the embryo had a slightly elongated suspensor compared with that of the S0 and S1 pre-embryos, while there was no obvious difference in the embryo itself (Fig. 1h and i). In S3 pegs, the basal embryo was typically in the globular stage (Fig. 1k and l). These results demonstrate that a rod-like pre-embryo forms as early as 4 days after fertilization. It remains inactive throughout stages S1 and S2 and is morphologically the same until enlargement of the pod after approximately 9 days of dark growth. The initiation of pod enlargement coincides with the re-activation of embryonic development.

### Sequencing data analysis

Using Illumina HiSeq™ 2000 sequencing technology, more than 12 million total reads were acquired from each sample. After removing adaptor sequences and low-quality reads, the number of total clean reads in each library used for subsequent analyses ranged from 11 to 12 million. Our results showed that the depth of sequencing was saturated for gene discovery (Additional file 3: Figure S1), indicating that the libraries represent the transcripts in each sample well. While the random RNA fragmentation ensured that read positions were evenly distributed across each gene, the coverage decreased at both 5ʹ and 3ʹ ends. Average gene coverage, the percentage of each gene covered by reads, for the twelve samples was 56 % (Additional file 3: Figure S2). Therefore, the sequencing data should accurately reflect gene expression and can be used for differential gene expression analysis. The use of RPKM values for determining gene expression levels eliminates any bias due to differences in gene length or sequencing discrepancies.

Approximately 8 million clean reads in each library matched the reference perfectly or with fewer than two mismatches. The number of clean reads in each library that uniquely matched reference genes ranged from 6.5 to 7.8 million, accounting for 57.35–64.04 % of each library (Table 1). If the criteria were set to include alignments with fewer than three mismatches to the reference genome, 9 million clean reads per library could be mapped to the genome. Among these, 7.5–8.3 million were unique matches, accounting for 65.16–68.30 % of each library (Additional file 4: Table S3). Due to differences in the genetic background and the limitations of annotation, approximately 60 % of the clean reads from each sample were uniquely mapped to reference genes, while 67 % were uniquely mapped to the reference genome.

**Table 1:** Summary of mapping result (mapping to reference genes)

Sample ID	Replication	Total clean reads	Total mapped reads	Perfect match	< =2 bp Mismatch	Unique match	Multi-position match	Total unmapped reads
S1-ER	1	11,449,209	7,690,109	5,586,546	2,103,563	6,965,933	724,176	3,759,100
	2	12,335,056	8,503,080	6,180,851	2,322,229	7,754,062	749,018	3,831,976
S1-BR	1	12,023,993	7,810,160	5,641,877	2,168,283	7,025,399	784,761	4,213,833
	2	12,257,878	8,266,679	6,035,647	2,231,032	7,515,875	750,804	3,991,199
S2-ER	1	11,446,627	7,898,285	5,754,463	2,143,822	7,206,860	691,425	3,548,342
	2	12,233,415	8,595,851	6,260,690	2,335,161	7,834,426	761,425	3,637,564
S2-BR	1	11,613,232	7,691,237	5,608,986	2,082,251	7,015,241	675,996	3,921,995
	2	11,917,155	8,085,290	5,876,588	2,208,702	7,289,596	795,694	3,831,865
S3-ER	1	11,802,971	8,121,699	5,958,191	2,163,508	7,391,705	729,994	3,681,272
	2	12,408,764	8,537,515	6,189,025	2,348,490	7,784,021	753,494	3,871,249
S3-BR	1	11,421,119	7,177,782	5,221,853	1,955,929	6,549,727	628,055	4,243,337
	2	11,762,649	7,523,532	5,479,311	2,044,221	6,784,105	739,427	4,239,117

### DEG analysis of pegs of different development stages

**Fig. 2:**
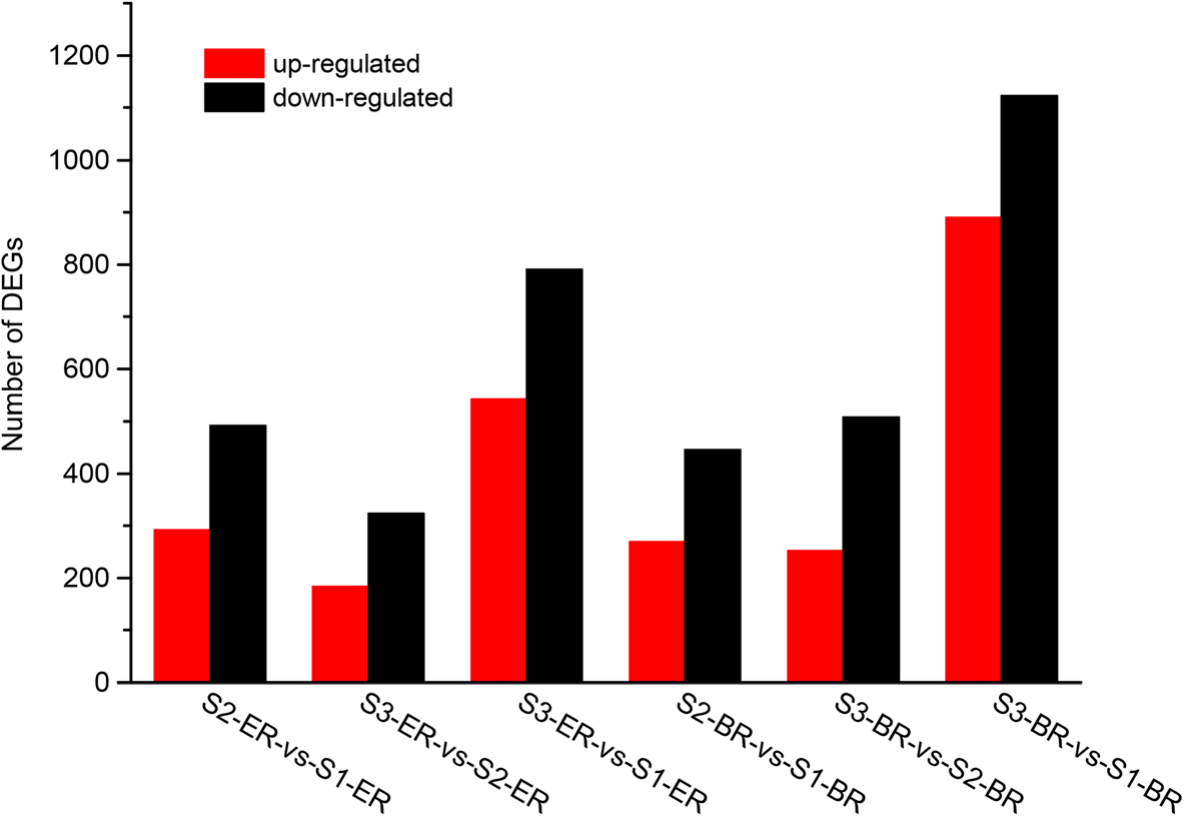
Pairwise comparative analysis of DEGs in three development stages and two regions of pegs. The number of up-regulated and down-regulated genes in the nine groups are indicated

**Fig. 3:**
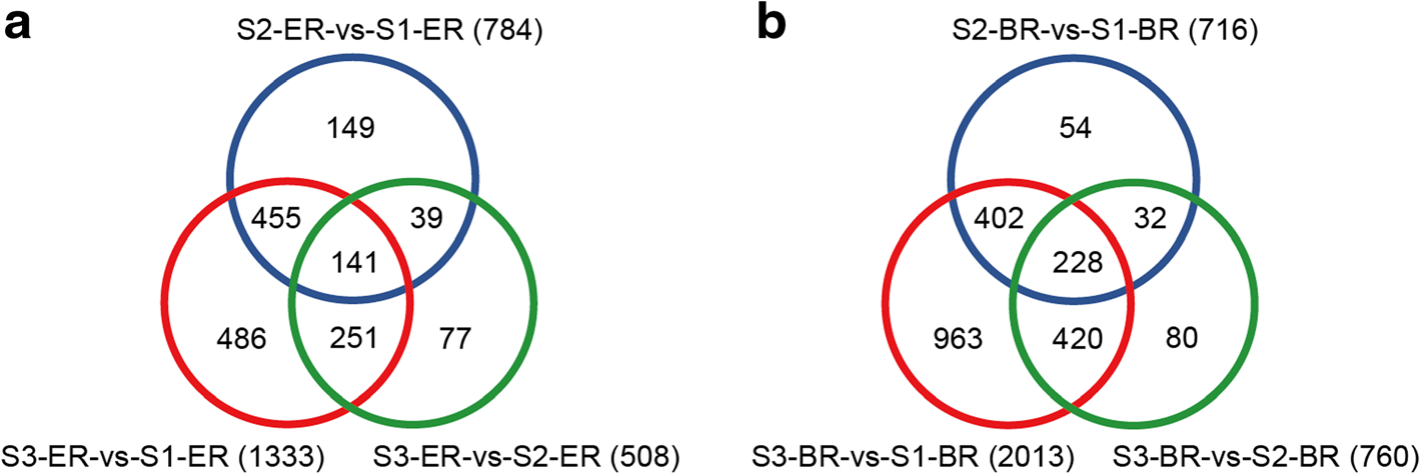
Number of DEGs in three development stages and two regions of pegs. For each group, the total number of differentially expressed genes is indicated. DEGs derived from the ER and BR of S1, S2, and S3 pegs are listed in (**a**) and (**b**), respectively

We compared the gene expression between S2 and S1, S3 and S1, and S3 and S2 in ER and BR samples independently for a total of six pairwise comparisons of DEGs. The correlations between the two replicates for each sample were higher than 96 % for all genes (Additional file 3: Figure S3). The total number of DEGs for each comparison is shown in Fig. 2, while additional information for each DEG is listed in Additional files 5, 6, 7, 8, 9 and 10: Tables S4–S9. Results showed that considerable changes occur at the transcriptional level during peanut peg elongation, soil penetration, and the initiation of pod enlargement. We discovered a large number of genes preferentially expressed during certain developmental stages (Fig. 3). These stage-specific DEGs, which are likely involved in early pod development in peanut, were used for further analyses.

### GO classification and KEGG enrichment analysis of DEGs

GO annotation of the DEGs from the six pairwise comparisons (S2-ER/S1-ER, S3-ER/S1-ER, S3-ER/S2-ER, S2-BR/S1-BR, S3-BR/S1-BR, and S3-BR/S2-BR), was used to classify genes into different sub-categories belonging to the three main GO categories: biological process, cellular component, and molecular function. In the biological process category, we identified three abundant sub-categories across all six comparisons: cellular process, metabolic process, and response to stimulus. Cell, cell part, and organelle were the main sub-categories identified in the cellular component category, while binding and catalytic activity dominated the molecular function category.

KEGG enrichment analysis allowed for the mapping of DEGs to different pathways. The top 20 pathways in each pairwise comparisons (mentioned above) are shown in Additional file 11: Figure S4. DEGs were mainly enriched in porphyrin and chlorophyll metabolism, photosynthesis, phenylpropanoid biosynthesis, phenylalanine metabolism, pentose and glucoronate interconversions, glycan degradation, glyoxylate and dicarboxylate metabolism, flavonoid biosynthesis, and carotenoid and secondary metabolic biosynthesis. Other enriched pathways included circadian rhythm, cutin, suberine and wax biosynthesis, nitrogen metabolism, starch and sucrose metabolism, ascorbate and aldarate metabolism, amino sugar and nucleotide sugar metabolism, and tyrosine metabolism.

### Gene expression changes of light signaling components

Genes encoding components of light signaling pathways exhibited differential expression when transitioning from aboveground (S1) to belowground (S2, S3) in both the ER and BR samples. In S2 and S3, genes encoding zinc finger protein LSD1, protein SPA1, phototropin, early light induced protein, and root phototropism protein 2 were down-regulated, both in the ER and BR, compared to expression in S1. In contrast, genes encoding CONSTANS and LHY were expressed more highly in S1-ER than that in S2-ER and S3-ER. While the expression of the COP1 gene was down-regulated in S2-ER compared to S1-ER, no differences were found between S2-BR and S1-BR. Similarly, the expressions of circadian clock-related *GIGANTEA* and circadian clock-associated *FKF1* were up-regulated in S3-BR compared to S1-BR, but no differences were detected between S3-ER and S1-ER.

The ER and BR of S2 and S3 pegs were completely white in color, indicating that the chlorophyll content was significantly reduced and that degeneration of photosynthesis had occurred. A number of genes that act in the chloroplast exhibited differential expression after peg soil penetration, including thylakoid membrane phosphoprotein 14 kDa, thylakoid lumenal protein, sedoheptulose-1,7-bisphosphatase, bisphosphate carboxylase/oxygenase activase 1, protochlorophyllide reductase, photosystem II CP43 chlorophyll apoprotein, photosystem I reaction center subunit, oxygen-evolving enhancer protein, and chlorophyll a-b binding protein. Most of these genes were down-regulated in both the ER and BR when comparing S2 to S1, with further expression reductions in S3.

### Expression changes in hormone-related genes

Five categories of plant hormone-related genes (auxin, gibberellin, cytokinin, ethylene, and ABA) were detected as DEGs in comparisons of S1, S2, and S3 in the ER and BR of pegs. In the ER, the indole-3-acetic acid-amido synthetase gene and auxin-induced protein gene were down-regulated in S2 when compared to S1. In the BR, the indole-3-acetic acid-amido synthetase gene was also down-regulated but the auxin-induced protein gene was up-regulated in S2 when compared to S1. In the ER, the expression levels of the tryptophan aminotransferase, PINOID, auxin-efflux carrier, auxin transporter protein, and auxin-induced protein genes were down-regulated while the auxin-repressed protein and indole-3-acetic acid-amido synthetase genes were up-regulated in S3 when compared to S1 and S2. In the BR, genes encoding tryptophan aminotransferase, PINOID, indole-3-acetic acid-amido synthetase, and auxin-transporter were down-regulated, and auxin-induced protein and auxin response factor genes were up-regulated in S3 vs. S1 and S2 pegs.

The expressions of several genes encoding gibberellin biosynthesis and signal transduction components were altered during peanut pod development. In the ER, the gibberellin 20 oxidase gene was up-regulated and several gibberellin-regulated protein genes and gibberellin 2-oxidase gene were down-regulated in S3 vs. S1 and S2. In the BR, gibberellin-regulated protein genes and the gibberellin 20 oxidase gene were down-regulated and the gibberellin 2-oxidase gene was up-regulated in S2 and S3 vs. S1. The expression of the gibberellic acid receptor gene was also up-regulated in the BR in S3 compared with that in S1.

ABA, cytokinin, and ethylene biosynthesis and signaling components were detected during early pod development. In the ER, the expression level of abscisic acid 8ʹ-hydroxylase (a key enzyme for ABA catabolism) was higher in S2 and S3 than in S1, and the abscisic acid receptor gene was down-regulated in S3 compared to that in S1 and S2. Similarly, in the BR, the gene encoding abscisic acid 8ʹ-hydroxylase was up-regulated and the abscisic acid receptor gene was down-regulated in S3 compared to that in S1. In the ER, cytokinin dehydrogenase genes were up-regulated in S2 and S3 vs. S1. These genes were only differentially expressed in the BR between S2 and S3, however, with a higher expression level in S3-BR. Ethylene-related DEGs were also identified in the ER among S1, S2, and S3, and the expression of the ethylene-responsive transcription factor and ACC oxidase was down-regulated in S3 vs. S1 and S2 in both the ER and BR. In addition, only ACC synthase was up-regulated in S2-ER compared to S1-ER, while ACC synthase and ACC oxidase were down-regulated in S2-BR compared to S1-BR. Genes encoding the ethylene receptor and EIN3-binding F-box protein were down-regulated in S3-BR compared to S1-BR.

### DEGs involved in pod enlargement in the ER

Besides phytohormone- and light signaling-related genes, other important DEGs were detected in the ER during initiation of pod swelling. These DEGs include transcription factor families, as well as cell wall relaxation and embryo-development related genes. In the ER, several transcription factor families were identified as DEGs in the transition from S1 to S3, including the WRKY transcription factor family, MYB transcription factor family, bHLH transcription factor family, and MADS transcription factor family. Genes encoding WRKY family members were up-regulated in S2-ER and S3-ER compared to S1-ER. MYB transcription factor genes were up-regulated in S3-ER compared to S1-ER and S2-ER. Genes encoding bHLH transcription factors were down-regulated while MADS box protein genes were up-regulated in S2-ER and S3-ER compared to S1-ER. In addition, the gene encoding GATA transcription factor 9 was down-regulated in S3-ER compared to S2-ER and S1-ER. The transcription factor TCP3, a member of the TCP family, plays an important role in embryogenesis and was found to be up-regulated in S3-ER vs. S1-ER and S2-ER.

Several genes that participate in cell wall biosynthesis and degradation exhibited altered expression levels during early pod enlargement. Xyloglucan endotransglucosylase/hydrolase protein (XHT), which is involved in the modification of cell wall components, is thought to be crucial for regulating plant growth and development. Several XHT-encoding genes were up-regulated in S2-ER and S3-ER vs. S1-ER and S2-ER, respectively. Endoglucanase genes were found to be up-regulated in S2-ER and S3-ER vs. S1-ER. Pectinesterase is a major component of the plant cell wall that breaks down pectin. Genes encoding pectinesterases were up-regulated in S2-ER and S3-ER vs. S1-ER. Pectate lyase, in contrast, was up-regulated in S2-ER compared to S1-ER but down-regulated in S3-ER compared to S1-ER and S2-ER. The genes encoding cellulose synthase were down-regulated in S2-ER and S3-ER vs. S1-ER. Several genes encoding expansin were down-regulated in S2-ER and S3-ER compared to S1-ER and S2-ER, respectively.

In the ER, genes encoding the LEA protein were down-regulated in S3 vs. S1 and S2. The YABBY gene family plays an important role in adaxial-abaxial polarity in lateral organ development. A YABBY gene was up-regulated in S2-ER and S3-ER compared to S1-ER and S2-ER, respectively. One gene encoding an amino acid permease was found to be down-regulated in S2-ER vs. S1-ER but up-regulated in S3-ER vs. S1-ER. The gene encoding cyclin-D was up-regulated in S2-ER and S3-ER compared to that of S1-ER. The expression level of homeobox-leucine zipper protein ATHB-6, which was found in previous research to be a negative regulator of the ABA response, was up-regulated in S2-ER and S3-ER compared to S1-ER. The gene encoding homeobox-leucine zipper protein AHTB-16 was similarly found to be up-regulated in S3-ER compared to S1-ER and S2-ER, while Knotted-1 was down-regulated in S3-ER vs. S1-ER and S2-ER. DA1, a negative regulator of seed and organ size, was found to be down-regulated in S3-ER vs. S1-ER.

### qRT-PCR validation of DEG results

**Fig. 4:**
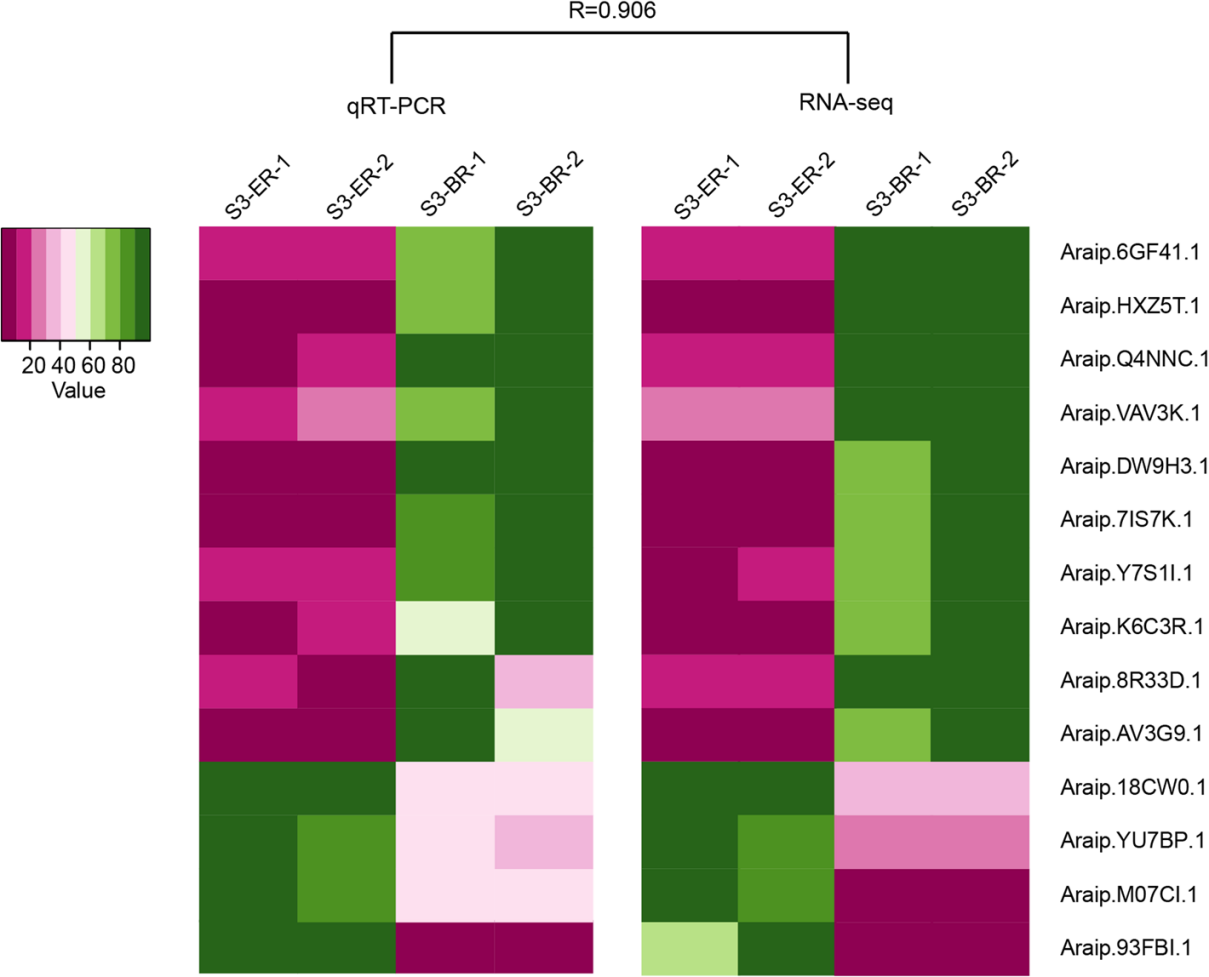
qRT-PCR verification of expression of selected genes. R = correlation between genes in S3-ER and S3-BR

To verify the RNA-seq results, we employed qRT-PCR to analyze the expression levels of 14 randomly selected DEGs in S3-ER and S3-BR. These genes were involved in phytohormone biosynthesis and signal transduction, embryo development, transcription factor regulation, and nitrate transport. The actin gene was used as an internal control. Gene-specific primers were designed using *Arachis ipaensis* nucleotide sequences. The expression patterns of these 14 genes were accordant with the RNA-seq data (Fig. 4). The coefficient of correlation between the qRT-PCR and RNA-seq data was 0.906, suggesting that the RNA-seq data were indeed credible.

## Discussion

Histological observations indicated that the peanut pre-embryo was morphologically similar from 4 days after pollination until 3 days after soil penetration (Fig. 1). When peanut pegs swell into 2–3 mm pods, the embryos are in the globular stage. This indicates that aerial pegs, white pegs without swelling, and enlarged pegs represent three important time points for peanut early embryo development. Two ovules are located 2–3 mm from the tip region of the pegs. Peg elongation is caused by the intercalary meristem, which is just behind the ovary [1]. If pegs are artificially hindered from soil penetration, the embryos in these pegs are eventually aborted [22]. The length of the pegs used in previous reports was approximately 10 mm, which reduced the ability to capture the changes in gene expression in the peanut pod. In this study, for more accurate analysis of regional gene expression, the peg was divided into two parts, the ER, or pre-embryo located region (0–3 mm from the peg tip), and the BR, or basal region (7 mm behind the peg tip). A large number of DEGs were identified in these two regions across the three developmental stages. Many of these genes are considered to be crucial for peanut peg elongation and pod development.

Previous research indicated that the processes of peanut peg elongation and pod swelling were controlled by light [8, 12, 13, 31, 32]. Excised pegs and intact pegs cease to elongate under dark conditions but continue elongation under continuous white, red, or blue light at a high luminous intensity [12, 13, 31, 32]. Compared to peg elongation, embryo development and pod enlargement respond in the opposite manner to light conditions. Darkness and far-red light stimulate peanut embryo development, while white, blue, and red light inhibit this process [8, 13, 31, 32]. These results suggest that light plays an important role in controlling the morphological changes of pod enlargement and peg elongation. The red/far-red light photoreceptor phytochrome is believed to be the regulator of embryonic development [8]. Several genes encoding phytochromes were detected in our transcriptome analysis; however, no differential expression of these genes was observed among S1, S2, and S3 in either the ER or BR. In *Arabidopsis*, phytochrome is regulated at the post-transcriptional level [33]. Degradation of phytochrome is mediated by COP1, which is a ubiquitin ligase that acts as a negative regulator of photomorphogenesis. In our earlier study, the expression level of COP1 was shown to be drastically down-regulated in S2 compared to that in S1 [21]. However, in this study, this down-regulation of COP1 expression was only detected in the ER. After soil penetration, phytochrome accumulates in the embryo and adjacent integument tissues [9], in accordance with the low expression level of COP1 observed in S2-ER. Blue light may provide two distinct functions impacting peg elongation, as response to high blue light intensity resembles that of red and white light, while low blue light illumination is considered to be far-red radiation [32]. The blue light receptor gene FKF1 was up-regulated in S3-BR, which suggests that the sensitivity of the BR to blue light was altered after peg soil penetration.

The contents of multiple endogenous plant hormones were considerably altered in S1, S2, and S3 pegs [13, 24]. In this study, among all phytohormone-related DEGs, the number of auxin-associated genes was the largest. Auxin plays a critical role in plant organ development, particularly in embryogenesis. In the two-step auxin biosynthesis pathway, tryptophan aminotransferase catalyzes the conversion of Trp into indole-3-pyruvic acid, and then IAA is produced by the flavin-containing monooxygenase YUCCA [34]. Both in the ER and BR, the tryptophan aminotransferase gene was down-regulated in S3 vs. S1. These results were in agreement with the low IAA content in subterranean pegs compared to aerial pegs [24]. However, the expression level of auxin-induced protein displayed opposite trends in the ER and BR: it was down-regulated in the ER and up-regulated in the BR after the pegs penetrated the soil. Similarly, genes encoding indole-3-acetic acid-amido synthetase also exhibited complementary changes in the ER and BR after peg soil penetration. In addition, genes for PINOID and auxin-efflux carrier, which alter auxin polar transport, also exhibited changes in expression level in the ER and BR during the transition from S1 to S3. Differences in the distribution of auxin may be a key reason for changes in peg morphogenesis. GA, an important plant hormone, regulates multiple aspects of plant growth and development. GA20-oxidase and GA2-oxidase catalyze the biosynthesis of active GA and inactivation of biological GA, respectively [35]. The expressions of the genes encoding these two enzymes displayed opposite patterns in the ER and BR. High expression of GA2-oxidase and low expression of GA20-oxidase in the BR may lead to low GA content, eventually causing peg elongation to cease after soil penetration. This is a novel discovery not found in our previous research.

**Fig. 5:**
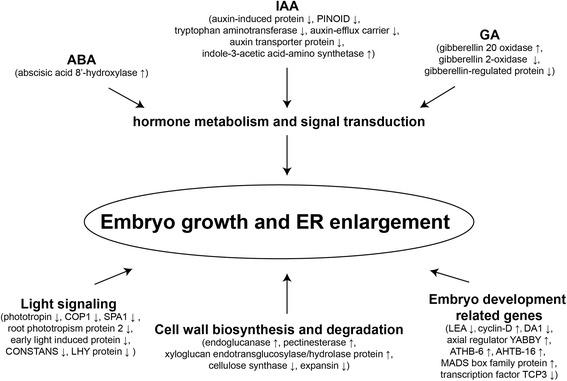
Identified DEGs from the ER of pegs and their functions during early pod development

In previous research, a wide range of differentially expressed genes were identified in aerial pegs and underground swelling pods [21]. In addition to light signaling and plant hormone-related genes, several embryonic development genes were identified in the ER of peanut pegs (Fig. 5). For example, the senescence-associated gene, LEA, and the embryo-abundant protein gene exhibited differential expression between aerial and subterranean pegs, in accordance with earlier research [22, 24]. Due to the dissection of the ER and BR in this study, we were able to more accurately detect transcriptome differences associated with embryonic development. The expression levels of LEA genes were lower in S2 and S3 pegs in the ER and higher in S1. An accumulation of LEA proteins in plants may enhance resistance to a stressful environment [36]. The high level of LEA gene expression in S1 pegs may reflect the fact that light is a stress factor to the peanut peg and the embryo inside it, while the low levels in the ER during pod swelling initiation may indicate that the environmental conditions are suitable for embryo development.

Members of the MADS box family were found to be highly expressed in the ER region of S2 and S3 pegs. MADS box transcription factors participate in multiple developmental processes during the plant life circle, including flower development, fruit ripening, early embryo development, seed pigmentation, and endothelium development [37–40]. The members of the transcription factor TCP family act redundantly to regulate the spatial expression of boundary-specific genes and control morphogenesis of the shoot meristem during embryogenesis [41]. Ectopic expression of boundary-specific genes results in multiple shoot meristems and inhibition of organ growth. A high expression level of TCP3 in S3-ER compared to that in S2-ER and S3-BR may suggest it plays a role in peanut early embryogenesis. Genes encoding YABBY family members and amino acid permease exhibited differential transcription levels in the ER before and after soil penetration. YABBY genes are mainly expressed in lateral organs (shoot apical and flower meristems) and are correlated with abaxial cell fate in *Arabidopsis* [42]. The lack of YABBY genes causes an extensive range of morphological changes in lateral organs [43, 44]. YABBY genes also play important roles in embryonic shoot apical meristem (SAM) formation. These genes are expressed on the abaxial side of cotyledon primordia in the globular stage [45]. The expression of a YABBY gene was down-regulated in the ER of S2 and S3 pegs. Amino acid permease, which functions as an amino acid transporter, plays an important role in the uptake of amino acids by the embryo [46–48]. The transcription of this gene is strongly induced before storage protein synthesis [49, 50]. Amino acid permease was first down-regulated in S2 pegs but subsequently up-regulated in S3 pegs. DA1, a ubiquitin receptor, negatively regulates seed and organ size by controlling the period of cell proliferation [51]. Reduced expression of the DA1 gene in the S2 and S3 ER may promote cell proliferation, contributing to the re-activation of embryonic development and increasing pod size.

Members of the HD-Zip protein family are involved in the regulation of plant developmental and environmental responses. ATHB-6, a class I HD-Zip transcription factor, is a negative regulator of the ABA signaling pathway [52]. ATHB-16, a paralog of ATHB-6, displayed similar changes in expression in the ER of S1, S2, and S3 pegs. Increased transcription of ATHB-6, ATHB-16, and abscisic acid 8ʹ-hydroxylase in the ER of S2 and S3 pegs may be related to the reduced ABA content of dark-grown pegs (Wang Xingjun, unpublished data). Pervious research showed that ABA inhibits peanut embryo growth under dark conditions, exhibiting a role similar to that of light [12].

## Conclusion

In this study, we independently analyzed differentially expressed genes in the pre-embryonic and basal regions of S1, S2, and S3 peanut pegs. Genes involved in light signaling transduction and phytohormone metabolism were found to be differentially expressed in the ER and BR. In addition, several genes involved in embryonic development were identified in the ER. These genes participate in the biosynthesis and decomposition of components of the cell wall, the polarity of embryo development, and amino acid transport. DEGs identified in the ER during the transition from S1 to S3 provide deeper understanding of the molecular mechanisms of light regulation of peanut embryo and pod development.

## Additional files

Additional file 1: Table S1. Gene-specific primers used in quantitative real-time PCR. (XLSX 10 kb) Additional file 2: Table S2. Verification of DEG results by qRT-PCR. Results show the mean Ct values of 14 randomly selected genes from S3-ER and S3-BR. RPKM of S3-ER and S3-BR denote RNA-seq results of DEG expression level. (XLSX 12 kb) Additional file 3: Figure S1. Sequencing depth was saturated for gene identification. Figure S2. Gene coverage analysis of S1-ER, S1-BR, S2-ER, S2-BR, S3-ER, and S3-BR. Figure S3. Correlation coefficient between the two biological replicates of each sample. (PPTX 1227 kb) Additional file 4: Table S3. Summary of mapping results to the reference genome. (DOCX 15 kb) Additional file 5: Table S4. Differentially expressed genes between S1-ER and S2-ER. (XLSX 158 kb) Additional file 6: Table S5. Differentially expressed genes between S2-ER and S3-ER. (XLSX 104 kb) Additional file 7: Table S6. Differentially expressed genes between S1-ER and S3-ER. (XLSX 258 kb) Additional file 8: Table S7. Differentially expressed genes between S1-BR and S2-BR. (XLSX 145 kb) Additional file 9: Table S8. Differentially expressed genes between S2-BR and S3-BR. (XLSX 152 kb) Additional file 10: Table S9. Differentially expressed genes between S1-BR and S3-BR. (XLSX 385 kb) Additional file 11: Figure S4. Scatter plot of top 20 KEGG pathways of six pairwise comparisons (S2-ER/S1-ER, S3-ER/S1-ER, S3-ER/S2-ER, S2-BR/S1-BR, S3-BR/S1-BR, and S3-BR/S2-BR). (TIF 1513 kb)
