# Erratum to: Comparative transcriptome analysis of basal and zygote-located tip regions of peanut ovaries provides insight into the mechanism of light regulation in peanut embryo and pod development

**DOI:** 10.1186/s12864-016-3181-5

**Published:** 2016-10-26

**Authors:** Ye Zhang, Pengfei Wang, Han Xia, Chuanzhi Zhao, Lei Hou, Changsheng Li, Chao Gao, Xingjun Wang, Shuzhen Zhao

**Affiliations:** 1 School of Life Sciences, Shandong University, Jinan, Shandong 250100 People’s Republic of China; 2 Biotechnology Research Center, Shandong Academy of Agricultural Sciences, Shandong Provincial Key Laboratory of Crop Genetic Improvement, Ecology and Physiology, Jinan, 250100 People’s Republic of China

## Erratum

The author list in the original publication [1] should have been displayed in the order below so that Shandong University comes first in the list of affiliations on the first page:

Ye Zhang^1,2^, Pengfei Wang^2^, Han Xia^2^, Chuanzhi Zhao^2^, Lei Hou^2^, Changsheng Li^2^, Chao Gao^1^, Xingjun Wang^1,2^* and Shuzhen Zhao^2^*

The author affiliations should have been as follows:

^1^ School of Life Sciences, Shandong University, Jinan, Shandong 250100, People’s Republic of China

^2^ Biotechnology Research Center, Shandong Academy of Agricultural Sciences, Shandong Provincial Key Laboratory of Crop Genetic Improvement, Ecology and Physiology, Jinan 250100, People’s Republic of China

## References

[1] Zhang Y (2016). Comparative transcriptome analysis of basal and zygote-located tip regions of peanut ovaries provides insight into the mechanism of light regulation in peanut embryo and pod development. BMC Genomics.

