# Supplementary figures and images for: Comparative transcriptome analysis of basal and zygote-located tip regions of peanut ovaries provides insight into the mechanism of light regulation in peanut embryo and pod development
Source: BMC Genomics. 2016 Aug 11;17:606. doi: 10.1186/s12864-016-2857-1 (PMC4982202; doi:10.1186/s12864-016-2857-1)

## Slide 1
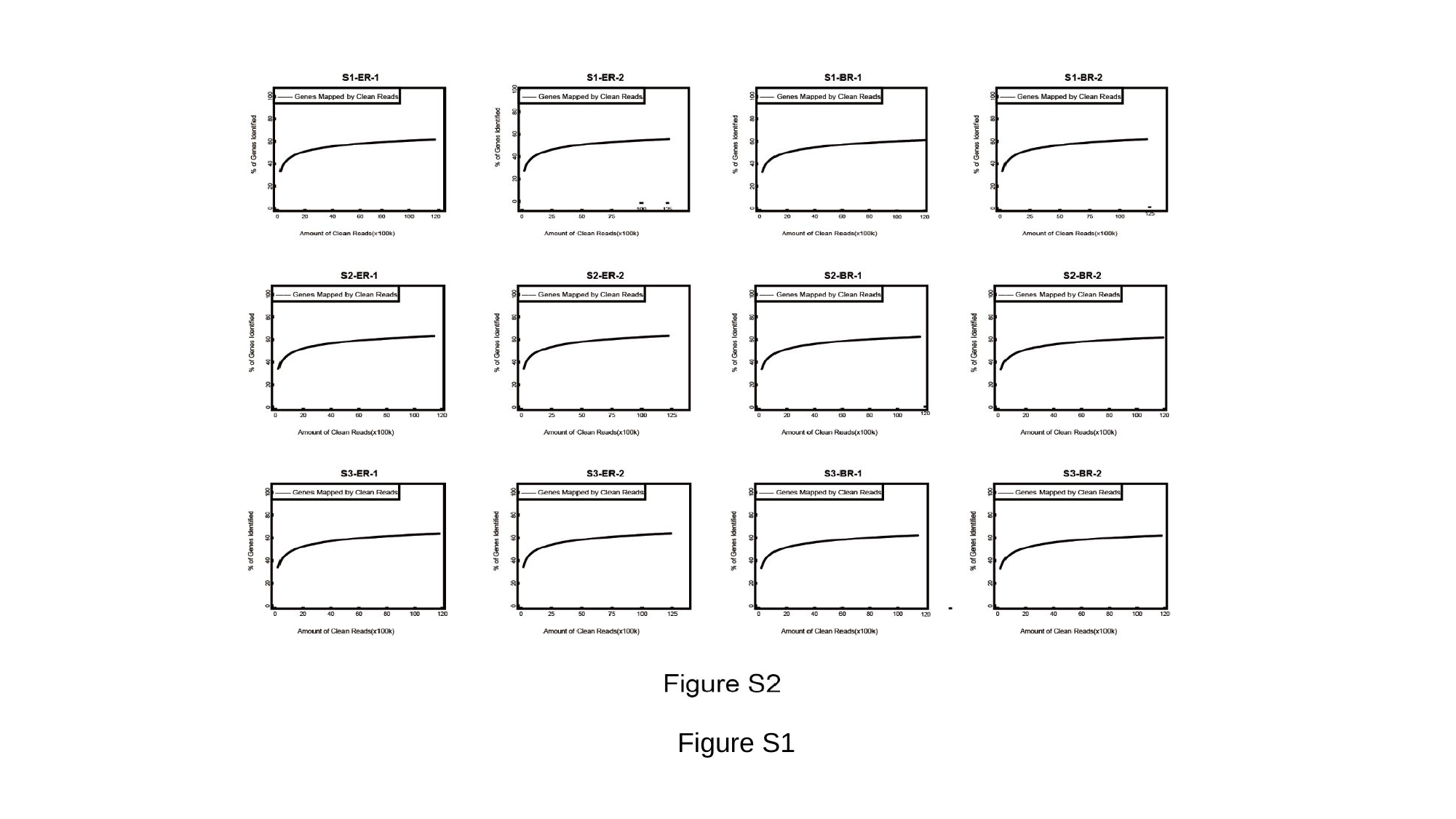

Figure S1

## Slide 2
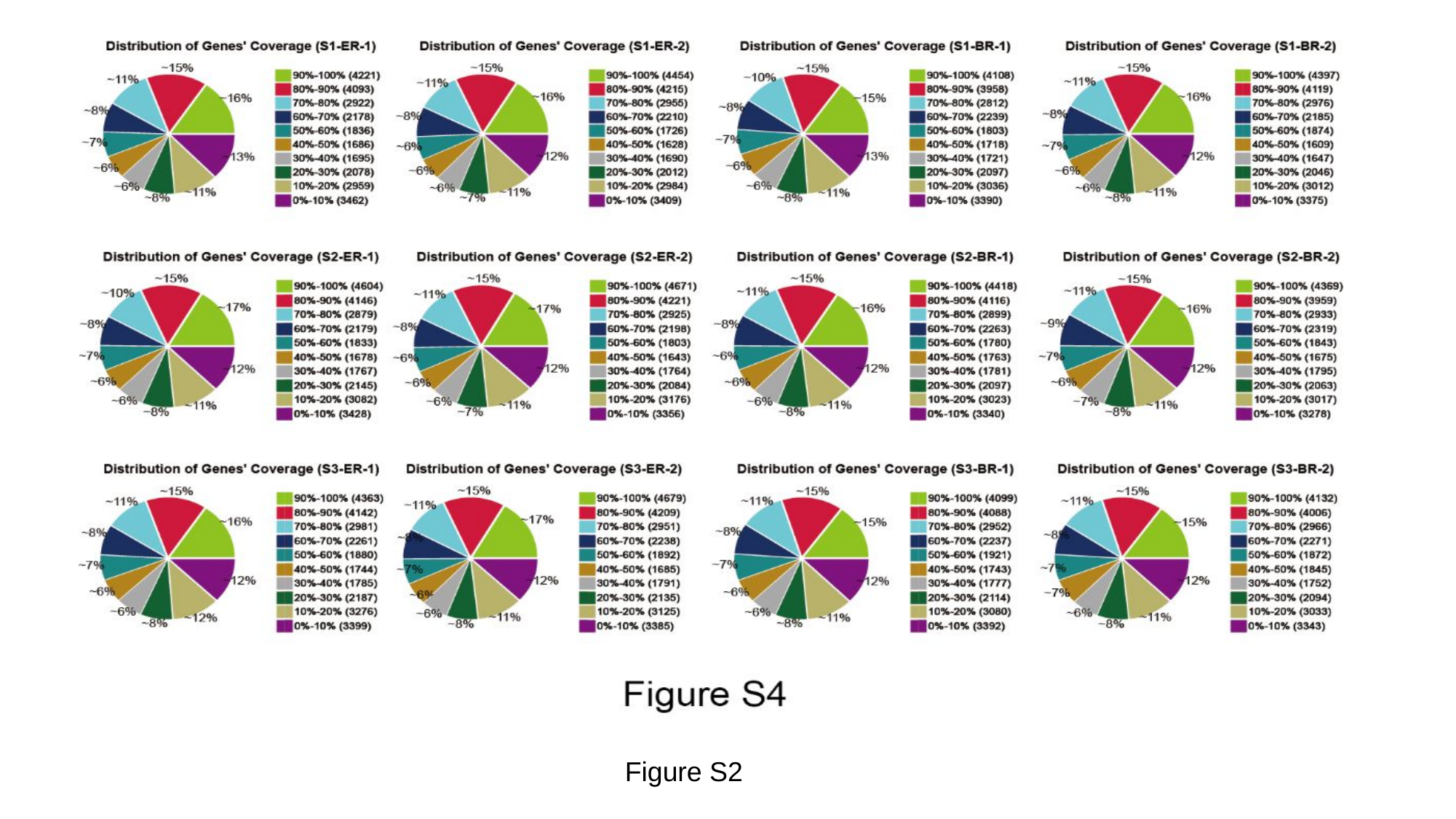

Figure S2

## Slide 3
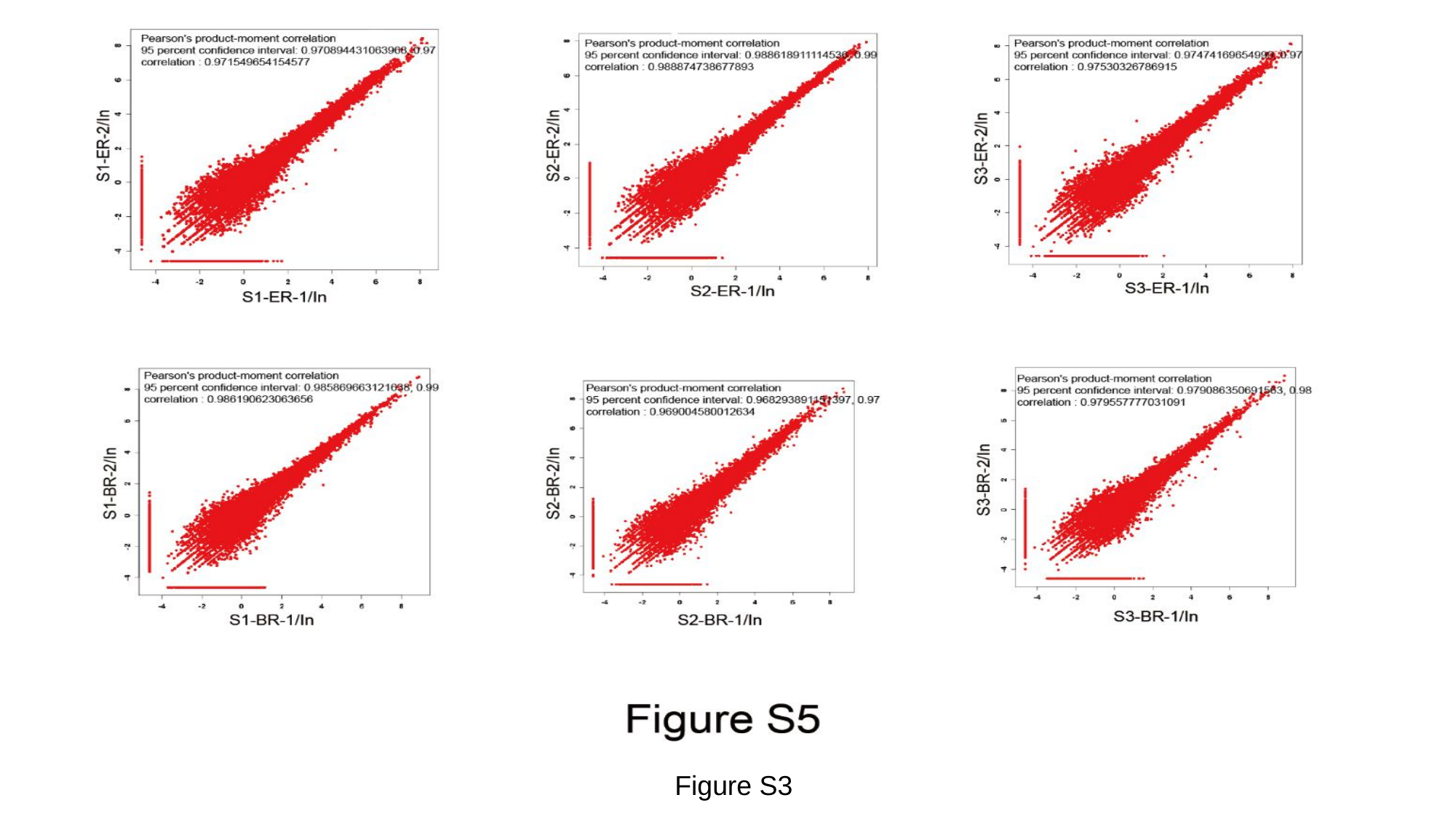

Figure S3

Supplement: Additional file 3: Figure S1. — Sequencing depth was saturated for gene identification. Figure S2. Gene coverage analysis of S1-ER, S1-BR, S2-ER, S2-BR, S3-ER, and S3-BR. Figure S3. Correlation coefficient between the two biological replicates of each sample. (PPTX 1227 kb) [file 12864_2016_2857_MOESM3_ESM.pptx]

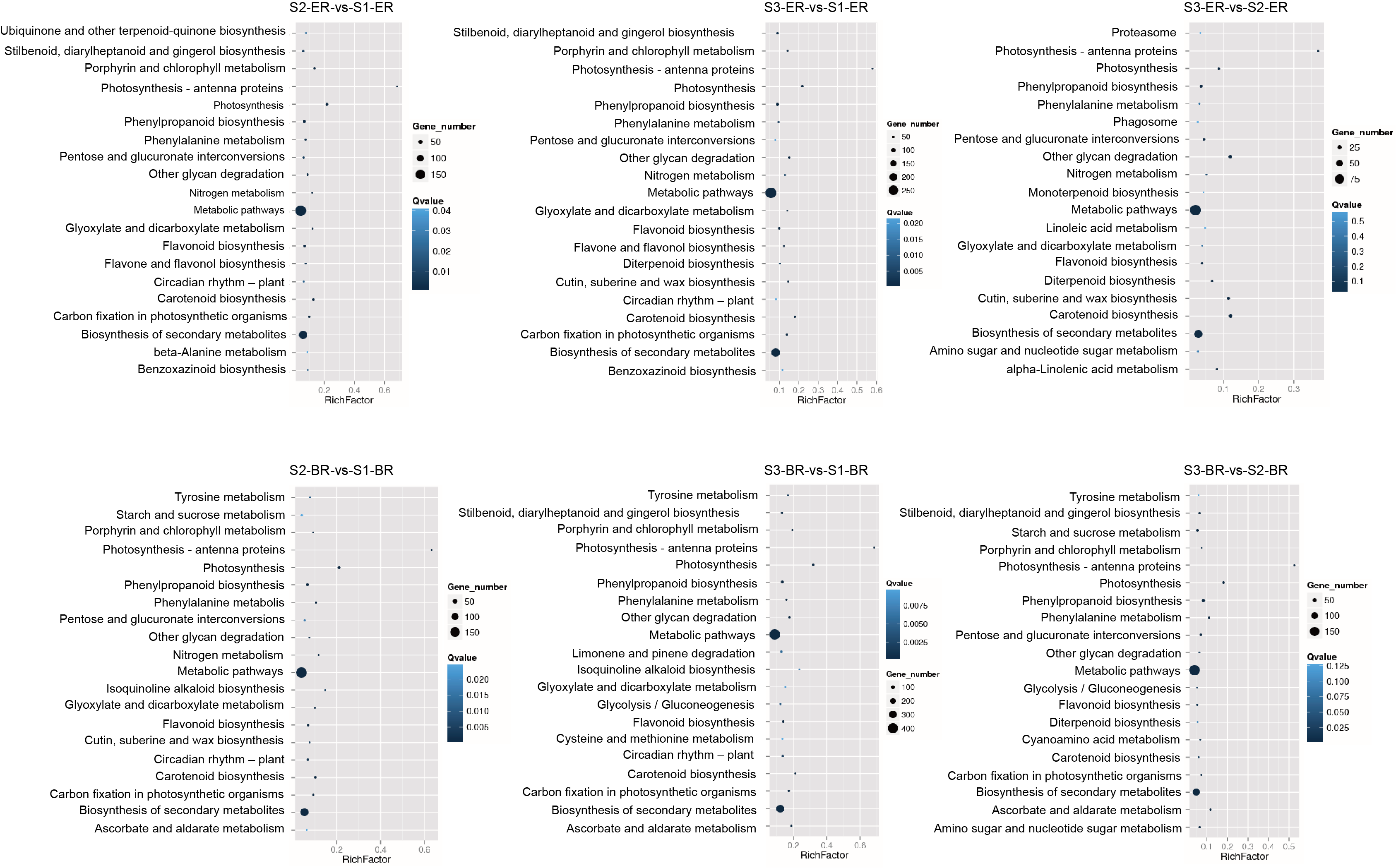

Supplement: Additional file 11: Figure S4. — Scatter plot of top 20 KEGG pathways of six pairwise comparisons (S2-ER/S1-ER, S3-ER/S1-ER, S3-ER/S2-ER, S2-BR/S1-BR, S3-BR/S1-BR, and S3-BR/S2-BR). (TIF 1513 kb) [file 12864_2016_2857_MOESM11_ESM.tif]
