# Supplementary material for: Comparative transcriptome analysis of basal and zygote-located tip regions of peanut ovaries provides insight into the mechanism of light regulation in peanut embryo and pod development
Source: BMC Genomics. 2016 Aug 11;17:606. doi: 10.1186/s12864-016-2857-1 (PMC4982202; doi:10.1186/s12864-016-2857-1)
Supplement: Additional file 4: Table S3. — Summary of mapping results to the reference genome. (DOCX 15 kb) [file 12864_2016_2857_MOESM4_ESM.docx]

**Table S3 Summary of mapping result (mapping to reference genome)**

| **Sample ID** | **Replication** | **Total Clean Reads** | **Total Mapped Reads** | **Perfect Match** | **<=3bp Mismatch** | **Unique Match** | **Multi-position Match** | **Total Unmapped Reads** |
| --- | --- | --- | --- | --- | --- | --- | --- | --- |
| S1-ER | 1 | 11,449,209 | 9,147,074 | 6,317,431 | 2,829,643 | 7,637,150 | 1,509,924 | 2,302,135 |
|  | 2 | 12,335,056 | 9,769,661 | 6,768,221 | 3,001,440 | 8,364,408 | 1,405,253 | 2,565,395 |
| S1-BR | 1 | 12,023,993 | 9,621,323 | 6,600,470 | 3,020,853 | 7,894,006 | 1,727,317 | 2,402,670 |
|  | 2 | 12,257,878 | 9,870,006 | 6,826,756 | 3,043,250 | 8,296,497 | 1,573,509 | 2,387,872 |
| S2-ER | 1 | 11,446,627 | 9,063,653 | 6,274,222 | 2,789,431 | 7,769,329 | 1,294,324 | 2,382,974 |
|  | 2 | 12,233,415 | 9,704,871 | 6,731,117 | 2,973,754 | 8,355,293 | 1,349,578 | 2,528,544 |
| S2-BR | 1 | 11,613,232 | 9,319,018 | 6,404,589 | 2,914,429 | 7,810,981 | 1,508,037 | 2,294,214 |
|  | 2 | 11,917,155 | 9,559,533 | 6,607,372 | 2,952,161 | 8,011,826 | 1,547,707 | 2,357,622 |
| S3-ER | 1 | 11,802,971 | 9,380,566 | 6,522,831 | 2,857,735 | 7,935,181 | 1,445,385 | 2,422,405 |
|  | 2 | 12,408,764 | 9,807,749 | 6,744,882 | 3,062,867 | 8,349,436 | 1,458,313 | 2,601,015 |
| S3-BR | 1 | 11,421,119 | 9,157,504 | 6,265,537 | 2,891,967 | 7,505,219 | 1,652,285 | 2,263,615 |
|  | 2 | 11,762,649 | 9,463,904 | 6,534,138 | 2,929,766 | 7,664,330 | 1,799,574 | 2,298,745 |
